# Virtual Hand Illusion in younger and older adults

**DOI:** 10.1177/20556683211059389

**Published:** 2021-12-07

**Authors:** Jennifer L. Campos, Graziella El-Khechen Richandi, Marge Coahran, Lindsey E. Fraser, Babak Taati, Behrang Keshavarz

**Affiliations:** 1 Department of Psychology, 7938 University of Toronto, Toronto, ON, Canada; 2 7961 KITE Research Institute - Toronto Rehabilitation Institute, University Health Network, Toronto, ON, Canada; 3 Department of Occupational Therapy, 7938 University of Toronto, Toronto, ON, Canada; 4 Department of Psychology, 7991 York University, Toronto, ON, Canada; 5 Department of Computer Science, 7938 University of Toronto, Toronto, ON, Canada; 6 Institute of Biomedical Engineering, 7938 University of Toronto, Toronto, ON, Canada; 7 Department of Psychology, 7984 Ryerson University, Toronto, ON, Canada

**Keywords:** Virtual Reality, aging, embodiment, avatar, rubber hand illusion, localization, proprioception, perception, multisensory

## Abstract

**Introduction:**

Embodiment involves experiencing ownership over our body and localizing it in space and is informed by multiple senses (visual, proprioceptive and tactile). Evidence suggests that embodiment and multisensory integration may change with older age. The Virtual Hand Illusion (VHI) has been used to investigate multisensory contributions to embodiment, but has never been evaluated in older adults. Spatio-temporal factors unique to virtual environments may differentially affect the embodied perceptions of older and younger adults.

**Methods:**

Twenty-one younger (18–35 years) and 19 older (65+ years) adults completed the VHI paradigm. Body localization was measured at baseline and again, with subjective ownership ratings, following synchronous and asynchronous visual-tactile interactions.

**Results:**

Higher ownership ratings were observed in the synchronous relative to the asynchronous condition, but no effects on localization/drift were found. No age differences were observed. Localization accuracy was biased in both age groups when the virtual hand was aligned with the real hand, indicating a visual mislocalization of the virtual hand.

**Conclusions:**

No age-related differences in the VHI were observed. Mislocalization of the hand in VR occurred for both groups, even when congruent and aligned; however, tactile feedback reduced localization biases. Our results expand the current understanding of age-related changes in multisensory embodiment within virtual environments.

## Introduction

A sense of embodiment allows us to maintain a feeling of ownership over our body, perceive the location of our body in space and experience agency over the movements of our body. This sense of embodiment is informed by integrating inputs from different sensory systems, including visual, proprioceptive, tactile and vestibular inputs (multisensory integration).^1–4^ The processing of these individual sensory inputs alone and in combination changes with older age. In addition to the well-known declines in individual sensory abilities in older adults, there is emerging evidence that both embodiment and multisensory integration may also change with older age. For example, there is some indication that older adults exhibit heightened multisensory integration (e.g. greater multisensory relative to unisensory effects) compared to younger adults,^5–12^ and that there are age-related changes to embodiment such as declines in sensorimotor processing and changes to motor imagery.^13–15^

### Embodiment in virtual reality

Understanding multisensory perceptions related to embodiment and how they change with older age might also have important implications for virtual reality (VR)-based research and applications (e.g. rehabilitation/therapeutics, entertainment, and training).^16,17^ For example, when reaching or grasping within a virtual environment, experiencing ownership and agency over your self-avatar and maintaining a precise and accurate estimation of the position of your arm/hand in space may have important implications (e.g. for task execution, learning). However, the technical limitations of VR can result in visual representations of the body and/or of the environment that are not veridical. For example, compressions of distance estimates are often reported due to inaccurate and/or conflicting depth cues presented in head-mounted displays, and sensory-motor lags can occur in VR.^18–20^ These effects may therefore result in spatial and/or temporal distortions that could affect perception and embodiment in virtual environments. It is also known that, generally speaking, age-related changes occur in the processing of visual cues including those important for depth perception such as binocular disparity and motion parallax,^21,22^ as well as age-related changes to sensorimotor processing^15^ that could uniquely influence older adults’ percepts within virtual environments. These age-related factors may also affect the experiences of ownership, agency and the perceptual spatial alignment of real and simulated features (e.g. location of the real limb vs. the location of one’s self-avatar within the virtual environment). Given that older adults may have much to gain from VR-based applications and that many applications developed specifically for older adults may benefit from a sense of embodiment (e.g. clinical and social interventions), understanding age-related differences in measures of VR-based perception and embodiment could be advantageous.

### Rubber hand illusion

A common paradigm that has been extensively used to examine the various factors that influence embodiment is the ‘Rubber Hand Illusion’ (RHI).^2,23–25^ In the classic RHI protocol, a rubber hand is placed on a table in a position shifted horizontally from the observer’s own real, hidden hand.^2,23–25^ The observer sees the rubber hand being stroked (visual input) while they feel their own hand being simultaneously stroked (tactile input). After a period of time, this temporally congruent visual–tactile feedback can create the illusion that the rubber hand belongs to the observer (increased self-reported sense of ownership) and can also create the perception that the occluded real hand has shifted in position towards the rubber hand (proprioceptive drift).^23,25,26^ This illusion often has a stronger effect when the visual–tactile feedback is temporally aligned (i.e. synchronous) than when it is temporally misaligned (i.e. asynchronous).^23^ When the spatial and/or temporal offset between the visual, tactile, and/or proprioceptive inputs becomes too large, the RHI does not occur.^27–29^ The range of spatial/temporal offsets within which the illusion still occurs, can be considered an individual’s spatial or temporal window of integration. It has been shown that individuals with larger temporal binding windows are more tolerant to greater visual-tactile temporal offsets, as evidenced by continued susceptibility to the rubber hand ownership illusion at greater offsets (but no changes in proprioceptive drift).^29^

While some studies have demonstrated that measures of self-reported ownership and proprioceptive drift are correlated, there is evidence that these two effects may be driven by dissociable processes.^30–34^ For instance, in a series of experiments, Rohde et al.^33^ showed that proprioceptive drift towards a false limb that was mis-localized in space relative to the real limb occurred even when no visual-tactile stimulation was applied. Notably, these experimental conditions did not elicit subjective feelings of ownership. While subjective ownership seems to require congruent visual-tactile cues, it has been suggested that drift reflects visual-proprioceptive integration specifically. It is posited that asynchronous stroking (the typical control condition for the RHI) actively inhibits this integration by creating a strong dissociation between visual and proprioceptive signals.^33^ Overall, the RHI provides a convincing demonstration of how multisensory integration processes contribute to embodiment and bodily localization, thereby making it a potentially valuable tool for assessing sensory integration across the lifespan.

### Virtual hand illusion

In more recent years, a VR-based version of the RHI, the ‘Virtual Hand Illusion’ (VHI), has been developed (e.g. Refs. 35–42). In younger adults, the VHI has been shown to replicate similar effects to those seen for the classic RHI, with the sensation of ownership of the virtual avatar arm and proprioceptive drift being associated with congruent visual-tactile stimulation. The VHI has been demonstrated using numerous types of virtual displays, from 2-D projected images onto flat surfaces^43^ and computer screens,^35^ to 3-D avatars presented stereoscopically on a large projection screen,^36–38^ and via head-mounted displays.^39,44^ For example, Slater and colleagues^37^ showed that subjective ownership and proprioceptive drift were stronger for a 3-D stereoscopically viewed virtual arm when a floating ball was shown tapping the virtual arm in synchrony with a felt touch, compared to asynchronous tapping. Other studies have shown that feelings of ownership can be induced over virtual avatars viewed from the first person perspective, despite differences in the avatar’s age,^45^ skin colour^46^ or apparent gender.^47^ Despite the growing body of literature on virtual body illusions, to our knowledge, no research has specifically investigated the effect of healthy ageing on these phenomena by comparing the percepts of older and younger adults.

### Age-related changes to the rubber hand illusion and virtual hand illusion

Until recently, little was known about whether age-related changes to sensory, motor, and/or cognitive functioning influenced the extent to which the RHI or VHI is observed in older adults compared to younger adults. There are several theoretical reasons to predict that older adults may be differentially susceptible to the RHI under different conditions. For example, the heightened multisensory integration often observed for older adults in other multisensory paradigms^11^ (for review) may lead to stronger visual-tactile coupling, which could enhance the ownership illusions. However, the larger temporal binding windows observed in older adults may also lead to smaller differences in responses to synchronous compared to asynchronous conditions, given that visual-tactile integration may still occur during larger temporal offsets (i.e. smaller relative illusion). Changes to basic sensory acuity (e.g. visual and/or proprioceptive) could also affect baseline visual and proprioceptive percepts of body position, even prior to visual-tactile coupling. However, in spite of these age-related factors, recent work has observed no age-related differences in the RHI when comparing older and younger adults.^26,48–50^ Only one study has demonstrated significant age-related differences in the classic RHI (greater effects during synchronous relative to asynchronous stroking), but these effects were only observed for the ownership illusion and not for proprioceptive drift.^51^ The few studies that have compared the RHI in younger and older adults were all conducted using the traditional, real-world version of the RHI. Thus, it remains largely unknown whether age-related differences that are specific to the VHI exist. Given the unique spatiotemporal features influencing sensorimotor interactions in virtual environments and the known age-related changes to these sensorimotor processes, it might be expected that greater age-related differences would be observed in the VHI compared to the RHI.

Even in the context of basic perceptual parameters and potential visual distortions in VR, very little is known about whether older adults experience these distortions similarly to what has been described in younger adults (e.g. spatial compression effects). As such, it is also important to compare older and younger adults’ baseline estimates of body position within virtual environments, even under conditions of visual and proprioceptive spatial alignment. In the case of the VHI, this would include establishing pointing estimates for the real hand (eyes open and closed) relative to the perceived position of the aligned and spatially shifted virtual hand at baseline (i.e. prior to any visual/tactile interactions). Measuring perceptual biases or distortions of body positions in virtual environments may also be important during VR-based applications requiring precise spatio-temporal calibrations.

### Current study

In this study we examined whether there are age-related differences in ownership and proprioceptive drift measures of the VHI (i.e. self-avatar presented via a head-mounted display). The main objectives of the study were to explore, (1) whether age-related differences are observed in the VHI, (2) whether any observed age-related differences are uniquely associated with ownership or proprioceptive drift measures, (3) whether there are any baseline differences in hand localization accuracy for the virtual hand relative to the real-world hand and (4) whether relative hand localization accuracy differs by age. To achieve this, proprioceptive localization measures were taken for the real hand at baseline (eyes open and closed), in the virtual environment (when aligned and when shifted relative to the real hand position) and following synchronous and asynchronous tactile stimulation. The subjective ownership questionnaire was completed following synchronous and asynchronous tactile stimulation. Overall, as virtual reality applications become increasingly more common, particularly in health care and training sectors, it is important to understand whether immersive virtual experiences are influenced by age-related changes in multisensory processing and embodiment.

## Methods

### Participants

Twenty one younger adults (9 females, 12 males; *M*_*age*_= 26.62, SD = 5.70) and 19 older adults (13 females, 6 males; *M*_*age*_=70.63, SD = 5.57) participated in this study. All participants were healthy, right-handed individuals between the ages of 18–35 (younger group) and 65+ (older group) with no self-reported history of neurological, psychiatric or musculoskeletal disorders or other major health conditions (e.g. diabetes). As is described below, sensory and cognitive screening measures were used to screen for cognitive impairment (Montreal Cognitive Assessment (MoCA)^52^) and visual acuity (ETDRS). Cutaneous tactile sensitivity (Semmes–Weinstein monofilament testing)^59^ was conducted to the inner thumb of each hand for older adults only, with all participants scoring within the normal range. Written informed consent was provided prior to completing the study, and participants were paid $10 for their participation. This research was approved by the University Health Network Research Ethics Boards (REB# 14-7793) and was performed in accordance with the ethical standards specified by the 1964 Declaration of Helsinki.

### Stimuli and apparatus

#### Sensory and cognitive assessments

The experiment took place in a quiet room at KITE – Toronto Rehabilitation Institute. Participants first completed a demographics and health history questionnaire to rule out any serious health conditions. Older participants also scored within the normal range of a standard visual acuity test (ETDRS) and completed the MoCA screening for mild cognitive impairment,^52^ all scoring above the cutoff of 26 (*M* = 28.35).

### Experimental materials and set-up

#### Tactile stimulator, VR system and motion tracking devices

The vibrotactile device consisted of five small vibrating motors providing tactile feedback that were strapped to the back and base of each finger, just above the knuckle. An Arduino board was used to interface between the motors and a computer to allow for turning the vibrators on and off (Arduino, Somerville, MA). Visual feedback was provided via an Oculus Rift head-mounted display (HMD; DK1 Facebook Technologies, LLC, Menlo Park, CA; 640x800 per eye resolution, 90° horizontal and vertical field of view, 60 Hz refresh rate). A Vicon motion capture system was used to track the HMD and the location of the hand in real-time (Vicon Motion Systems, Oxford, UK). The real-time tracking of both the HMD and the hand enabled the relative localization of the two, such that a virtual hand could be rendered with an offset relative to the user’s point of view. In the experimental conditions involving a shifted representation of the virtual hand (misaligned, synchronous and asynchronous conditions), the offset was set to 14 cm to the right of the participant’s real hand. The Vicon system consisted of three motion capture cameras that tracked the 3-D location of three retroreflective markers fixed to a rigid body grasped by the hand and five retroreflective markers affixed to the HMD, from which the 3-D location and orientation of the HMD was calculated. Prior to each experiment the motion capture system was calibrated via a standard calibration procedure.

The virtual environment was developed using the Unity game engine (Unity Technologies, San Francisco, CA). The avatar was presented from a first-person perspective and consisted only of the left arm and hand (no other part of the body visible), which remained in a stationary position. No customizations were made to adjust the appearance of the avatar for each participant (e.g. gender, skin colour). The virtual environment consisted of a largely empty room with a table. The visual representation of the tactile stimulus was a red ball approximately 2 cm in diameter. The ball moved on a downward trajectory towards the avatar’s hand and when it made visual contact with the base of each finger, the vibrotactile device was activated for 100 ms. The spatial and temporal alignment depended on the condition; for the synchronous it was aligned, but for the asynchronous condition it was misaligned. In the asynchronous condition, the temporal misalignment between the visualization of the contact and the felt vibration was randomly chosen from between 0.2 and 1.35 s. This also resulted in randomized spatial misalignments between the seen and felt location of the stimulus across the fingers associated with the temporal offsets.

### Dependent measures

Two main measures were recorded: hand localization/proprioceptive drift and subjective ownership. First, hand localization was measured using grid paper that was fixed to the underside of the table directly below the participant’s left hand. Participants were handed a felt-tipped marker in their right hand and asked to reach underneath the table and place a dot on the grid paper in a location aligned with the tip of the index finger on their real left hand. For each experimental condition, this pointing procedure was repeated three times. The average of the three pointing trials was used for statistical analyses. Second, a 7-item Ownership Questionnaire (adapted from^23^) was administered (see Table 1). Reponses were coded on a Likert scale ranging from 1 (strongly disagree) to 7 (strongly agree). It was expected that an illusion of ownership would result in higher scores for items 1–3 but not for items 4–7.

**Table 1.** Results of the mixed factorial ANOVAs for each item on the Ownership Questionnaire.

		Main effects					
		Condition			Age		
#	Item	*F*	*p*	η_p_^2^	*F*	*p*	η_p_^2^
1	I felt as if the artificial hand was my own	10.858	.002**	.222	1.267	.267	.032
2	The touching of the artificial hand felt just like an actual touch	30.948	< .001**	.449	0.536	.468	.014
3	It felt as though the artificial hand was in the same position as my real hand	8.147	.007**	.177	0.076	.785	.002
4	It seemed as if the touch I was feeling came from somewhere between my own hand and the artificial hand	0.046	.831	.001	2.608	.151	.064
5	It seemed as if I might have more than two hands	2.440	.127	.060	0.569	.455	.015
6	The artificial hand began to resemble my real hand, in terms of shape, skin tone, freckles or some other visual features	1.111	.299	.028	0.015	.903	.000
7	My own hand felt artificial	1.069	.308	.027	0.217	.644	.006

### Procedure

Every participant completed six different pointing conditions; (1) baseline pointing with eyes open, (2) baseline pointing with eyes closed, (3) VR visually aligned, (4), VR visually misaligned, (5) synchronous stimulation and (6) asynchronous stimulation. Conditions 1–4 were always performed in the same order for every participant and were performed once before Condition 5 (synchronous) and again before Condition 6 (asynchronous) (or vice versa). The repetition of these four conditions was used to carefully quantify any practice or carryover effects and to ensure repeatability of these ‘baseline’ conditions. There were no significant differences on the first and second trials for these conditions and therefore they were averaged. Conditions 5 and 6 (synchronous and asynchronous) were performed one time each and were counterbalanced for order across participants. In the real room, participants were seated at a height-adjustable table positioned just above waist height and were assisted in putting on and adjusting the HMD (display black) and the vibrotactile stimulation devices (the HMD was briefly removed to complete Condition 1 – see below). Their left hand always remained still on the table and their right hand rested at their side out of view. The six different conditions were the following:

1. *Baseline Pointing with Eyes Open to Real Arm:* After positioning their left hand and with full vision available (no HMD), participants completed the pointing task three times. This provided a measure of pointing performance under full sensory conditions and was used as an indicator of how well participants could complete the pointing task and whether there were any biases.
2. *Baseline Pointing No Vision:* The HMD was donned but remained blank. Participants again performed the pointing task three times. This provided a measure of pointing performance and general precision of proprioceptive body localization estimates when no visual information was available during pointing.
3. *Pointing with Virtual Arm Aligned:* The HMD was turned on and the room and the avatar were visible. The screen went blank and three trials of the pointing task were completed. This provided a measure of perceived arm position when simulated visual inputs were aligned with proprioceptive inputs. It was intended to quantify any perceptual biases present in the VR simulation.
4. *Pointing with Virtual Arm Misaligned:* The HMD was turned on and the visible avatar arm was shifted 14 cm rightward from the position of the real arm. The screen went blank, and three trials of the pointing task were completed. This provided a measure of proprioceptive shift (relative to pointing response in Condition 3 when aligned).
5. *Synchronous Visual-Tactile (SYNCH) Condition:* With their real left hand motionless and while looking directly at the virtual hand, the virtual ball visibly contacted the fingers and was accompanied by a simultaneous vibrotactile sensation at the same location on their real hand. Synchronous visual-tactile stimulation began with the thumb and continued until reaching the pinky finger; the order was then reversed. This pattern was repeated for 5-min after which the screen went blank. The pointing task was repeated again three times. This provided a measure of whether any proprioceptive drift (relative to Condition 4; i.e. the VHI) was observed. Participants also then completed the Ownership Questionnaire.
6. *Asynchronous Visual-Tactile (ASYNCH) Condition:* This condition was the same as the synchronous condition, with the exception that random temporal incongruencies were introduced between the vibrotactile input applied to the real hand and the corresponding image of the virtual object on the virtual hand. Three pointing responses were completed, followed by the Ownership Questionnaire.

Between the SYNCH and ASYNCH conditions participants were given a 5-min break, during which time they were asked to take off the HMD and complete a simple maze drawing task. The break was intended to readjust participants back to baseline in order to reduce any order effects and to reduce fatigue from wearing the HMD.

### Study design and analyses

The experiment consistent of a 2 (age group: younger, older) x 6 (condition: baseline pointing eyes open, baseline pointing eyes closed, VR visually aligned, VR visually misaligned, SYNCH, ASYNCH) mixed factorial design with age group as a between-subjects factor. Statistical analyses were performed using the Statistical Package for Social Sciences (SPSS; IBM) and the software R. A priori significance level was set to alpha = .05.

## Results

### Ownership questionnaire

**Figure 1.**
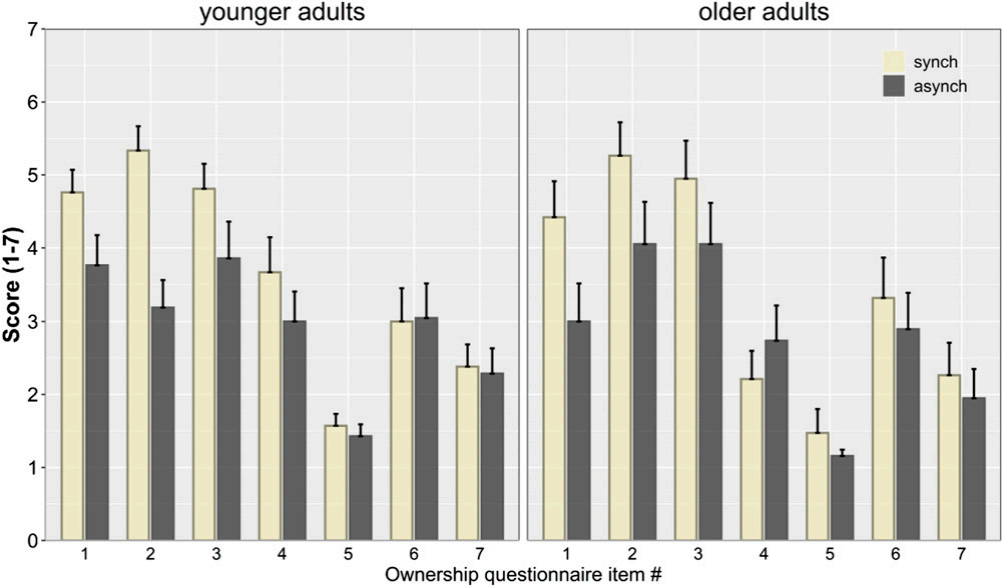
Averaged scores for each of the ownership questionnaire items (see Table 1 for item number descriptions) for younger adults (left panel) and older adults (right panel) after synchronous and asynchronous tactile stimulation. Error bars represent SEM.

Averaged scores for each of the ownership questionnaire’s items separated by condition are shown in Figure 1. To analyze the difference in ownership ratings between the SYNCH and ASYNCH condition, mixed factorial ANOVAs including the within-subject factor condition (SYNCH, ASYNCH) and the between-subjects factor age group (younger, older) were calculated for each of the questionnaire’s items.^53^ The results for the main effects of condition and age group are shown in Table 1, with significant main effects of condition observed for the predicted items (but not the control items), and no main effect of age group or interaction between condition and age group (*p*’s > .11).

### Proprioceptive Drift and Hand Localization Estimates

**Figure 2.**
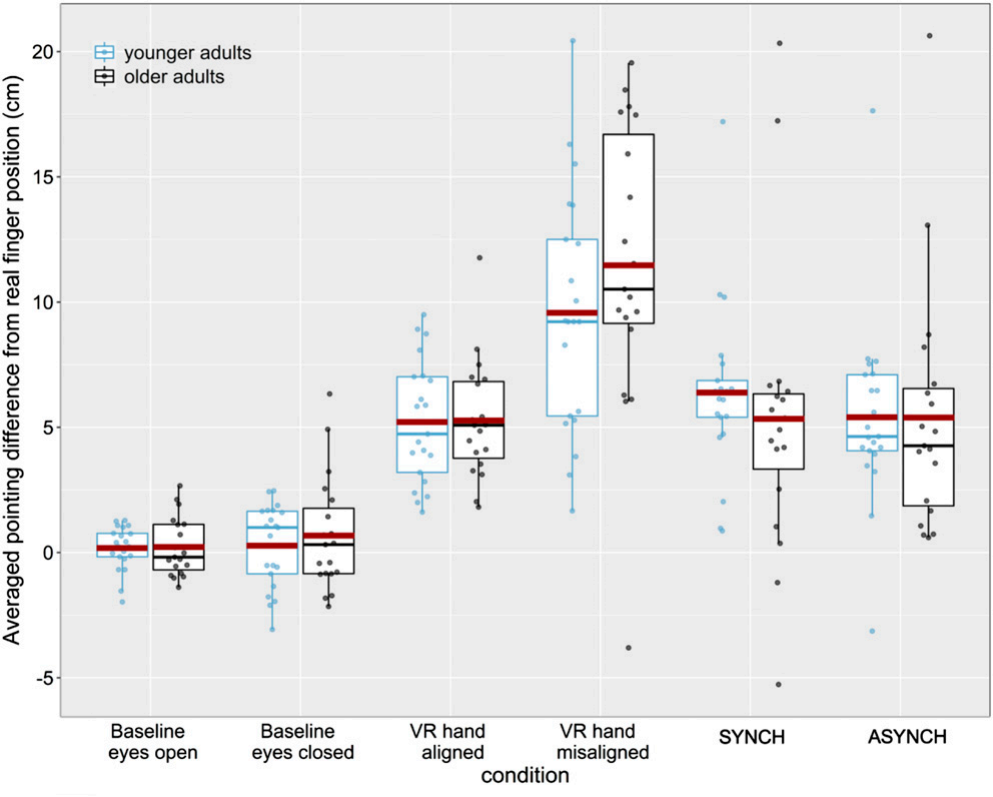
Boxplots showing the distribution of pointing results in relation to the actual position of the left index finger (cm) for older and younger adults at baseline (eyes open; eyes closed), with the VR hand visible (aligned with real hand; shifted towards the participant’s real body by 14 cm), and after tactile stimulation for both synchronous and asynchronous conditions. Positive values indicate a horizontal shift towards the right. Dots represent individual data points, and the red line indicates the mean.

Average scores for the pointing tasks for each condition and for both age groups (younger and older) are shown in Figure 2^1^.

To compare the pointing task measures after each experimental condition, a mixed factorial ANOVA including the within-subject factor condition (baseline eyes open pointing, baseline pointing eyes closed, VR aligned, VR misaligned, SYNCH, ASYNCH) and the between-subjects factor age group (younger, older) was calculated. Results showed a significant main effect of condition, *F*(5, 190) = 86.564, *p* < .001, η_p_^2^ = .695. Post-hoc comparisons (Bonferroni corrected) showed significant differences between baseline measures (both eyes open and eyes closed) and all of the other four conditions (*p*’s < 001). Additionally, pointing measures when the VR hand was visible but misaligned were significantly larger than when the VR hand was visible and aligned and after the synchronous and asynchronous conditions (*p*’s < 001). No significant differences between asynchronous and synchronous conditions were observed for either age group. No other comparisons were significant, nor was there a main effect of age group, or a condition by age group interaction.

Spearman correlations between each item of the ownership questionnaire and the average pointing results after tactile stimulation were calculated for the synchronous and asynchronous condition separately. For the synchronous condition, a significant positive correlation showed between questionnaire item #5 (‘It seemed as if I might have more than two hands’) and the results of the pointing task, *r* = .384, *p* = .015. All other correlations were not significant (r’s ranging from .009 to .264). For the asynchronous condition, significant positive correlations were found between the results of the pointing task and questionnaire items #3 (‘It felt as though the artificial hand was in the same position as my real hand’), *r* = .371, *p* = .018, and the questionnaire item #5 (‘It seemed as if I might have more than two hands’), *r* = .396, *p* = .011. All other correlations were not significant (*r*’s ranging from −0.030 to .165).

## Discussion

In this study, we examined whether there are age-related differences in the VHI as evidenced through ownership and proprioceptive drift measures. The VHI was observed with respect to the ownership measures, with higher ratings of ownership in the synchronous relative to the asynchronous conditions. However, for proprioceptive drift measures, there were no differences between synchronous and asynchronous conditions. No meaningful or predicable correlations were observed between ownership ratings and proprioceptive drift measures. These patterns of results were the same for younger and older adults, indicating no age-related differences in the VHI. In terms of hand localization accuracy, as expected, there were high levels of accuracy observed for the baseline real hand conditions (eyes open and closed). However, when the virtual hand was aligned with the real hand at baseline, localization accuracy was biased, indicating a visual mislocalization of the virtual hand in space, suggesting that it was perceived to be closer to the body mid-line than it was physically. When the virtual hand was intentionally displaced from the real physical hand to create a visual-proprioceptive misalignment, localization measures were also larger in the same direction of the displacement, indicating a strong influence of visual inputs on localization, even prior to visual-tactile interactions. However, once tactile inputs were introduced (either synchronous or asynchronous), localization responses were shifted back towards the aligned estimates. Importantly, these effects of visual-proprioceptive-tactile interactions on hand localization across baseline and experimental conditions did not differ for younger and older adults.

In general, these results are consistent with those of previous studies reporting no age-related differences in the traditional RHI,^26,48–50^ (but see 51). and expand these findings to demonstrate that similar outcomes are observed for the VHI. It is, however, possible that while no large age-related differences were observed in the current study, more subtle differences could be evidenced with a larger sample size. It was also confirmed that, at baseline, older adults demonstrated high proprioceptive localization accuracy of their hand with eyes open and closed, and their estimates did not differ significantly from younger adults. Finally, these results confirm that, for both younger and older adults, perceptual discrepancies occur between the real physical position of the limb and the visually perceived location of the limb in VR, even during aligned and spatially congruent conditions. The introduction of tactile stimulation appears to shift the weighting of localization estimates back towards the proprioceptively represented location, away from the visually shifted location.

One of the hallmark features of the RHI has been differences in ownership and proprioceptive drift in synchronous stimulation compared to asynchronous stimulation. The assumption here is that the integration of visual and tactile inputs is enhanced when the temporal rule of integration is met (temporally congruent) compared to when it is not met (temporally incongruent). Previous research has demonstrated that even during visual-tactile asynchronous conditions, the RHI can still be observed as long as the temporal conflicts that are introduced are within one’s own temporal binding window. For example, Costantini et al.^29^ demonstrated that the wider an individual’s temporal binding window is (as measured using a visual-tactile simultaneity judgement task), the more tolerant they are to greater levels of asynchrony introduced in the RHI. Interestingly, these associations were seen for subjective ownership judgements (visual-tactile integration), but not for proprioceptive drift measures (visual-proprioceptive integration). In other literature, it has been shown that older adults demonstrate greater bimodal (relative to unimodal) benefits, wider visual-tactile spatial binding windows and require longer temporal offsets to accurately perceive the temporal order of visual-tactile pairs of stimuli relative to younger adults.^7,54,55^ Taken together, it is possible that the degree of visual-tactile incongruency introduced in the current study may not have been large enough to observe age-related differences if this incongruency did not exceed older adult’s window of integration. It might be predicted that older adults would be more tolerant than younger adults to larger asynchronies as evidenced through ownership illusions that persist with longer temporal offsets. However, if this was the case, older adults may also exhibit smaller relative differences between synchronous and asynchronous conditions during smaller temporal incongruencies. The one study that compared asynchronous and synchronous conditions in older and younger adults^51^ demonstrated a *lower* ownership illusion in older compared to younger adults and no age-related differences in proprioceptive drift. Future research could strategically manipulate the degree of asynchrony during the VHI and/or measure individuals’ visual-tactile temporal binding window to determine whether these factors lead to observable age-related differences under particular conditions.

A feature of the VHI that is unique compared to the traditional RHI is that unintentional spatio-temporal distortions may occur, which could potentially disrupt body localization, embodiment, and/or sensori-motor interactions.^18,20^ The results of the current study demonstrated some evidence of spatial compression consistent with what has previously been reported in VR. Specifically, when the virtual hand was aligned with the real hand at baseline, localization estimates were perceived to be closer to the body mid-line than the actual physical position of the arm. Importantly, these results were similar for younger and older adults, indicating that older adults were not differentially affected by the interpretation of the visual cues in the virtual environment in a way that uniquely affected their localization estimates. Of course, the extent to which these types of spatial biases are observed is likely related to many factors, including the characteristics of VR device used (e.g. field of view, stereoscopic capabilities), the nature of the content (i.e. number of monocular and binocular depth cues available in the virtual environment) and the degree of interaction allowable within the virtual environment (e.g. updated head/body movements, cause-and-effect with virtual objects), which could allow for sensory-motor calibrations.^18,56–58^ It is also important to consider the extent to which particular VR-based applications are reliant on absolute sensory alignments and/or veridicality with real world metrics, or whether consistent relative associations are acceptable. Future research and applications should consider the relevance of these spatio-temporal biases for project-specific interpretation and implementation.

While proprioceptive localization measurements were included for baseline conditions (i.e. eyes open and closed real hand and eyes open VR), the ownership questionnaire was not administered after these trials. It would, however, be interesting to compare measures of ownership of the virtual hand to the measures of ownership of the real hand. Kanayama et al.^42^ compared responses to the RHI and the VHI in a group of healthy younger adults and reported that ownership and proprioceptive drift measures were generally larger for the VHI than the RHI. In a previous study,^26^ we also measured the RHI in a group of younger and older participants using a very similar paradigm to the one used in the current VHI study. Descriptive between-group comparisons between that previous study and the current study suggest that ownership ratings for the synchronous condition were higher in the RHI compared to the VHI and that proprioceptive drift was significantly greater in synchronous compared to asynchronous conditions only for the RHI, but not for the VHI. Therefore, further studies examining the differences in embodiment in the real world versus VR as evidenced by comparing between real and virtual versions of the illusion would help to provide clarity.

In summary, this study confirmed that the lack of age-related effects largely observed previously for the RHI is also observed in the VHI. Older adults demonstrated similar localization estimates of baseline, aligned and misaligned hand locations as younger adults, demonstrating that they were not differentially affected by VR-specific perceptual factors. Interestingly, the implementation of tactile feedback moved perceptual estimates of location away from the misaligned visual inputs, back towards the real physical position of the limb. Future research could consider how individual differences (e.g. temporal binding window, sensory acuity) and technology-related factors (e.g. field of view, depth cues, interactivity) contribute to age-related differences in embodiment within VR.

## Notes

1 All statistical analyses were also performed for the standard deviation of the pointing task to investigate age differences with respect to variability. No differences in variability were found between younger and older adults, *F*(1, 38) = 0.465, *p =* .500, η_p_^2^ = .012.
